# Frequency of autoimmune diseases in children and adolescents with type 1 diabetes mellitus

**DOI:** 10.3389/fendo.2026.1776403

**Published:** 2026-06-29

**Authors:** Melek Yaman Ortakoylu, Elmas Nazli Gonc, Alev Ozon, Nurgun Kandemir, Ayfer Alikasifoglu

**Affiliations:** 1 Department of Pediatrics, Faculty of Medicine, Hacettepe University, Ankara, Türkiye; 2 Division of Pediatric Endocrinology, Department of Pediatrics, Faculty of Medicine, Hacettepe University, Ankara, Türkiye

**Keywords:** autoimmune comorbidities, autoimmune diseases, childhood and adolescence, screening, type 1 diabetes mellitus

## Abstract

**Background:**

Children and adolescents with type 1 diabetes mellitus (T1D) are at increased risk of developing additional autoimmune diseases. However, data on the frequency, spectrum, and timing of these comorbidities in long-term pediatric cohorts remain limited. This study aimed to evaluate the prevalence, clinical characteristics, and temporal relationships of autoimmune diseases in children and adolescents with T1D, as well as to identify factors associated with their occurrence.

**Methods:**

This retrospective study included 639 children and adolescents with T1D who were followed at a tertiary pediatric endocrinology center between January 2004 and October 2018. Demographic, clinical, laboratory, and autoantibody data were reviewed. Autoimmune diseases were identified through routine annual screening and clinical follow-up. Temporal relationships between T1D and autoimmune disease diagnoses were analyzed. Multivariable logistic regression was used to assess factors associated with additional autoimmune diseases.

**Results:**

At least one additional autoimmune disease was identified in 120 subjects with diabetes (18.8%). The most frequent comorbidities were autoimmune thyroiditis (AIT, 12.7%) and celiac disease (CD, 5.9%), followed by vitiligo (1.3%), autoimmune gastritis/pernicious anemia (0.15%), and autoimmune hepatitis (0.15%). Female sex was independently associated with the presence of additional autoimmune diseases (OR = 1.61, 95% CI 1.07–2.41, *p* = 0.022). Most autoimmune diseases were diagnosed either concurrently with or after T1D onset, clustering within the first two years. A moderate positive correlation was observed between age at diagnosis of T1D and age at diagnosis of AIT (*r* = 0.586, *p* < 0.001), with older age at diagnosis of T1D associated with a shorter interval between T1D onset and the diagnosis of AIT. Similarly, age at diagnosis of T1D was strongly correlated with age at diagnosis of CD (ρ = 0.723, *p* < 0.001).

**Conclusion:**

Autoimmune comorbidities, particularly AIT and CD, are common in pediatric T1D, with most diagnoses occurring within the first two years around diabetes onset. These findings support intensified and closer screening—especially during the early disease course and in female subjects with diabetes—to enable earlier detection and improved long-term management. This study provides valuable epidemiological data from Türkiye and contributes to global pediatric T1D literature.

## Introduction

Type 1 diabetes mellitus (T1D) ranks among the most prevalent chronic disorders in childhood, characterized by insulin deficiency stemming from damage to pancreatic beta cells. In 90–95% of cases, this deficiency is a result of autoimmune destruction of beta cells (1). It is well-established that individuals with autoimmune disorders are predisposed to developing other autoimmune conditions (2). Those with T1D face an elevated risk of developing various autoimmune and related disorders, including chronic autoimmune thyroiditis (AIT), celiac disease (CD), Addison’s disease, autoimmune gastritis, autoimmune hepatitis (AIH), dermatomyositis, vitiligo, and myasthenia gravis.

Autoimmune thyroiditis emerges as the most prevalent autoimmune condition linked to diabetes, affecting 17–30% of individuals with type 1 diabetes. The prevalence of T1D alongside CD ranges from 1.6% to 16.4% globally (3, 4). In individuals with T1D, concomitant AIT or CD may complicate clinical management through their effects on thyroid function, growth, nutritional status, gastrointestinal symptoms, glycemic variability, and hypoglycemia risk, particularly when these conditions remain unrecognized (5). Therefore, it is imperative to thoroughly investigate the presence of other autoimmune diseases during the follow-up of individuals with T1D.

This study aimed to ascertain the frequency and characteristics of autoimmune diseases in children and adolescents diagnosed with T1D who were followed between January 2004 and October 2018 at the Department of Pediatric Endocrinology of Hacettepe University Children’s Hospital.

## Methods

Medical records and database entries of 1027 individuals with T1D who received care at a tertiary university hospital and reference center between January 2004, and October 2018 were carefully reviewed. Among these, 639 cases with confirmed diabetes autoantibodies were included in the analysis, while 388 cases were excluded due to inaccessible files or missing antibody results.

Demographic data including date of birth, gender, height, and body weight measurements, along with height standard deviation score (SDS), body mass index (BMI), and BMI z-score were recorded. Parameters related to T1D such as age at diagnosis, duration of follow-up, and the presence of diabetes-specific autoantibodies were documented. Routine annual screening was systematically performed for AIT and CD in all individuals during follow-up. Other autoimmune comorbidities, including Addison’s disease, autoimmune gastritis/pernicious anemia, AIH, and vitiligo were not systematically screened annually in all individuals; rather, their evaluation was clinically driven. For additional autoimmune diseases, the timing of diagnosis was recorded to evaluate their temporal relationship with the onset of T1D.

For cases exhibiting AIT, the time interval between diagnosis of T1D and the onset of thyroiditis, along with pertinent laboratory data including anti-thyroglobulin antibody (Anti-Tg) and anti-thyroid peroxidase (Anti-TPO) antibody levels, thyroid ultrasonography findings, and thyroid function tests at diagnosis were recorded. Similarly, specific autoantibody positivity including tissue transglutaminase immunoglobulin A (IgA), tissue transglutaminase immunoglobulin G (IgG), anti-endomysium IgA, anti-gliadin IgA, and anti-gliadin IgG) was documented. The small intestine biopsy findings, classified according to the MARSH classification system, were documented for individuals diagnosed with CD following upper gastrointestinal endoscopy. Selective IgA deficiency cases were identified through recorded IgA values during follow-up.

Height SDS values were calculated using the Centers for Disease Control and Prevention data (6), while BMI z-scores were determined using the lambda–mu–sigma method (7). Anti–glutamic acid decarboxylase (Anti-GAD) antibody levels were measured using IMMUNOTECH brand IM3650 kits and the immunoradiometric assay (IRMA) method with iodine-125, with threshold of >1 U/mL considered positive. Islet cell-associated phosphatase 2 (IA-2) antibody levels were determined using IMMUNOTECH brand IM3652 kits and the IRMA method with iodine 125, also with >1 U/mL considered positive. Anti-insulin antibody (IAA) levels were analyzed using BioSource brand KIP0091 kits and the IRMA method with iodine 125, with positivity defined as >6%. Individual data for diabetes-specific autoantibody tests conducted outside our hospital were retrieved from the respective files.

The diagnosis of AIT was based on a combination of clinical and laboratory findings indicative of thyroiditis, including elevated levels of Anti-Tg and Anti-TPO antibodies at values of at least four times the reference range. Additionally, features such as thyroid enlargement or atrophy, hypoechoic appearance, nodularity with echogenic septa, heterogeneous vascular distribution, and hypo vascularity on ultrasound further supported the diagnosis (8). The diagnosis of CD relied on the presence of specific antibodies along with histological confirmation via small intestine biopsy. For less frequent autoimmune conditions, diagnoses were recorded based on documented specialist evaluations and disease-specific clinical, laboratory, serological, histopathological, and/or imaging findings available in the medical records. Addison’s disease was evaluated in clinically suspected individuals using adrenal function tests, including serum cortisol and adrenocorticotropic hormone levels, and adrenal autoantibodies when available. Autoimmune gastritis/pernicious anemia was defined according to the presence of compatible hematological and gastrointestinal findings together with vitamin B12 deficiency and/or autoimmune markers such as parietal cell or intrinsic factor antibodies, when available. The individual with autoimmune gastritis/pernicious anemia had been diagnosed and followed at an external center, and this diagnosis was recorded based on the available medical records. Autoimmune hepatitis was defined based on compatible liver biochemistry, autoimmune serology, exclusion of other causes, and histopathological confirmation when performed. Vitiligo was diagnosed clinically by dermatological examination. The individual with AIH was recorded based on the diagnosis documented by the pediatric gastroenterology department in the medical records; detailed diagnostic data were not available in the study database. These less frequent conditions were not screened routinely in all individuals but were investigated when clinically indicated.

The study protocol was approved by the Hacettepe University Non-Interventional Clinical Research Ethics Committee (approval number: 16969557-1381; approval date: October 10, 2017). Given the retrospective design of the study and the use of anonymized medical record data, the requirement for written informed consent was waived by the ethics committee.

### Statistical methods

Statistical analyses were performed using the Statistical Package for the Social Sciences (SPSS) for Windows, version 20 (IBM SPSS Inc., Chicago, IL, USA). The normality of continuous variables was assessed using the Kolmogorov-Smirnov test and visual inspection of histograms. Normally distributed continuous variables were presented as mean ± standard deviation, whereas non-normally distributed variables were expressed as median (minimum –maximum). Categorical variables were summarized as numbers and percentages. Comparisons between two independent groups were performed using the Student’s *t*-test for normally distributed variables and the Mann–Whitney *U* test for non-normally distributed variables. Comparisons among more than two groups were conducted using one-way ANOVA or the Kruskal–Wallis test, as appropriate. *Post-hoc* analyses were applied where necessary. The homogeneity of variances was assessed using Levene’s test. Associations between categorical variables were evaluated using the chi-square test or Fisher’s exact test when expected cell counts were small. Correlation analyses were performed to evaluate the relationship between age at diagnosis of T1D and age at diagnosis of AIT and CD, using Pearson’s or Spearman’s correlation coefficients as appropriate. Linear regression analysis was applied to examine the association between age at diagnosis of T1D and age at diagnosis of AIT, with model fit assessed using the coefficient of determination (R²). Multivariable logistic regression analysis was used to assess factors associated with the presence of additional autoimmune diseases. Results were reported as odds ratios (ORs) with 95% confidence intervals (CIs). Multicollinearity was evaluated using variance inflation factors. All analyses were two-tailed, and a p-value < 0.05 was considered statistically significant.

## Results

A total of 639 children and adolescents with T1D were included in the study. Of these, 312 (48.8%) were male and 327 (51.2%) were female. There was no significant difference in the gender distribution of individuals with diabetes (p = 0.553). The mean age at diagnosis was 7.93 ± 4.0 years (range, 0.5 –17.66 years). The mean follow-up duration of individuals with T1D was 3.9 ± 3.7 years. In 120 cases (18.8%), at least one additional autoimmune disease accompanied diabetes. Of these individuals, 73 (60.8%) were female, 47 (39.2%) were male, resulting in a female-to-male ratio of 1.55. The prevalence of autoimmune disease accompanying diabetes was significantly higher in females compared to males (p = 0.019). Among the 120 individuals, 12 (10.0%) had been diagnosed with an autoimmune disease before the onset of T1D, 46 (38.3%) had a concomitant autoimmune disease at the time of diagnosis of T1D, and 62 (51.7%) developed an autoimmune disease after diagnosis of T1D during follow-up, with a median interval of 2.88 years (range, 0.08–15 years). Autoimmune thyroiditis was present in 81 cases (12.7%), CD in 38 cases (5.9%), vitiligo in 8 cases (1.3%), autoimmune gastritis/pernicious anemia in 1 case (0.15%), and AIH in 1 case (0.15%). A total of 8 individuals were diagnosed with multiple autoimmune diseases. Among the eight individuals, four had AIT and CD, three had AIT and vitiligo and one had AIT, CD and vitiligo. There was no significant difference in the age at diagnosis of autoimmune disease (including the age at diagnosis of T1D) between individuals with and without additional autoimmune diseases (p = 0.275) or between individuals with a single autoimmune disease and those with multiple autoimmune diseases (p = 0.920).

At least one diabetes-specific autoantibody was detected in all cases. Anti-GAD antibody was positive in 493 cases (77.2%), IA-2 antibody in 407 cases (63.7%), and anti-insulin antibody in 199 cases (31.1%). A single autoantibody was found in 257 cases (40.2%), two autoantibodies in 303 cases (47.4%) and three autoantibodies in 79 cases (12.4%) (**Table 1**). The distribution of single versus multiple diabetes-specific autoantibodies did not differ significantly between female and male individuals or among individuals without, with a single, or with multiple additional autoimmune diseases, nor did the prevalence of additional autoimmune diseases differ between individuals with a single versus multiple autoantibodies (p = 0.811, p = 0.969 and p = 0.879, respectively). There was no significant difference in the age at diagnosis of autoimmune disease between individuals with single and multiple diabetes-specific autoantibodies (p = 0.630).

**Table 1:** Demographic, clinical, and laboratory characteristics of the cases included in the study.

Parameters	Cases (total: 639)
Gender (Male/Female)	312 (48.8%)/327 (51.2%)
Age at diagnosis of T1D	
Median	7.91 (0.5-17.66)
Mean	7.93 ± 4.0
Follow-up duration of T1D	3.93 ± 3.7
Height SDS median	0.27 (-4.67-5.52) (N:590)
BMI Z-score median	0.18 (-6.37-6.75) (N:578)
Anti-GAD antibody positivity	493 (77.2%)
IA-2 antibody positivity	407 (63.7%)
Anti-Insulin antibody positivity	199 (31.1%)
Single antibody positivity	257 (40.2%)
Two antibodies positivity	303 (47.4%)
Three antibodies positivity	79 (12.4%)

BMI, body mass index, GAD, glutamic acid decarboxylase, IA, islet antigen, N, Number of individuals, SDS, standard deviation score.

The frequency of a positive family history of autoimmunity did not differ between individuals with and without additional autoimmune disease [15.0% (18/120) vs 12.3% (64/519); p = 0.43]. There was no significant difference in the frequency of positive family history between individuals with a single autoimmune comorbidity and those with multiple autoimmune comorbidities (p = 0.657). However, individuals with a family history of autoimmunity were diagnosed with T1D at a younger age than those without [6.75 years (1–15.75) vs 8.08 years (0.5–17.6); p = 0.008]. Eight (1.2%) individuals have selective IgA deficiency, and two of them were diagnosed with AIT.

In the multivariable logistic regression analysis, female sex was independently associated with the presence of additional autoimmune diseases (OR = 1.61, 95% CI 1.07–2.41, p = 0.022). The number of diabetes-related autoantibodies, family history of autoimmunity, selective IgA deficiency, age at diagnosis of T1D, and age at diagnosis of autoimmune disease were not significantly associated. Multicollinearity diagnostics were acceptable (all VIFs < 5) (**Table 2**).

**Table 2:** Multivariable logistic regression analysis for predictors of additional autoimmune diseases in children with T1D.

Variable	OR [Exp(B)]	95% CI for OR	p-value
Female sex (ref:male)	1.60	1.07-2.41	0.022
Multiple vs single autoantibodies	1.09	0.45-2.63	0.839
Family history of autoimmunity	0.85	0.48-1.51	0.584
Age at diagnosis of T1D	0.98	0.93-1.03	0.497
Age at diagnosis of autoimmune disease	0.97	0.93-1.03	0.303
Selective IgA deficiency	1.31	0.26-6.65	0.747

CI, confidence interval, Exp(B), exponentiation of the B coefficient, OR, odds ratio, T1D, type 1 diabetes mellitus.

**Figure 1:**
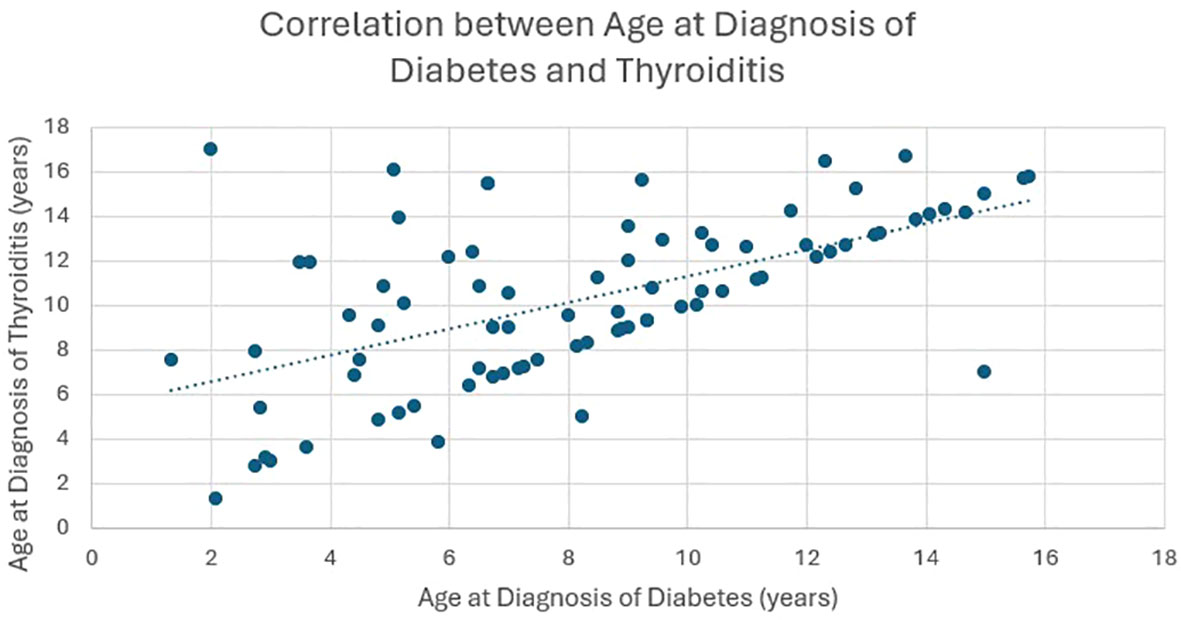
Correlation between age at diagnosis of diabetes and age at diagnosis of thyroiditis.

Among 81 cases with AIT, 67% were females and 33% were males, making the condition twice as common in females as in males (p = 0.001). Six individuals (7.5%) were diagnosed with AIT a median of 1.4 years (range, 0.16–8.0 years) before the diagnosis of diabetes, 34 individuals (42.5%) were diagnosed at the time of diagnosis of diabetes, and 40 individuals (50%) were diagnosed with AIT a median of 3.4 years (range, 0.25–15 years) after the diagnosis of diabetes. **Figure 1** shows a moderate, statistically significant positive correlation between age at diagnosis of diabetes and age at diagnosis of thyroiditis (r = 0.586, p < 0.001). This suggests that individuals diagnosed with diabetes at an older age tend to be diagnosed with thyroiditis at an older age as well. Further linear regression analysis confirmed that the age at diagnosis of diabetes was a significant predictor of the age at diagnosis of thyroiditis (F (1, 79) = 41.38, p < 0.001), with the model explaining approximately 34.4% of the variance (R² = 0.344). In linear regression, each 1-year increase in age at diagnosis of diabetes was associated with a 0.594-year increase in age at diagnosis of AIT (unstandardized B = 0.594, p < 0.001). There was no statistically significant difference in the age at diagnosis of diabetes between individuals with and without thyroiditis (p = 0.349).

**Figure 2:**
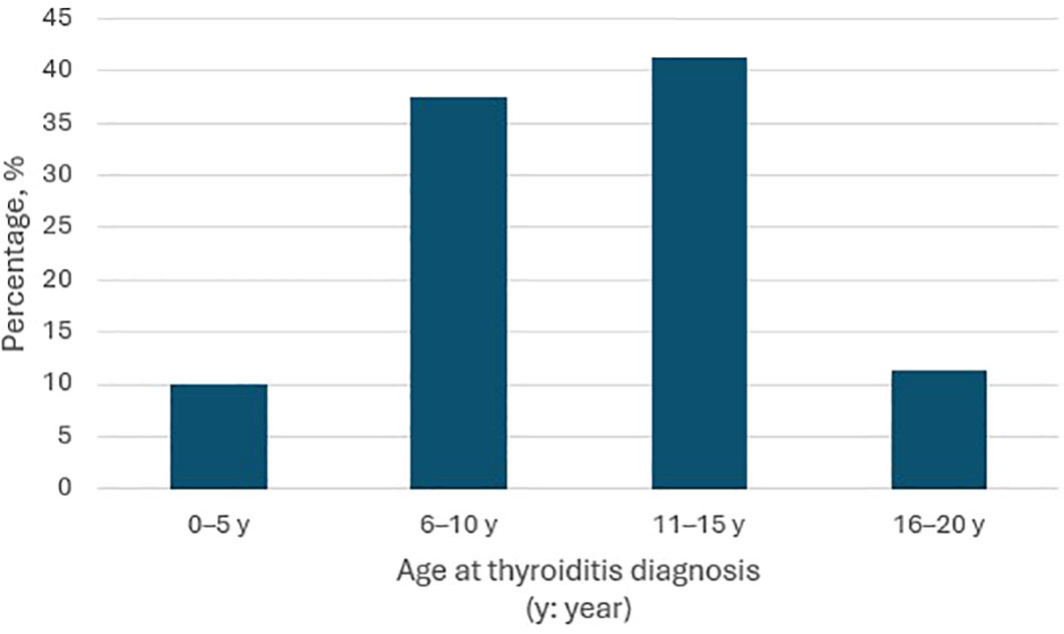
The frequency of thyroiditis by age.

The mean age at diagnosis of AIT was 10.30 ± 3.75 years. There was no statistically significant difference in the age at the time of diagnosis of thyroiditis between girls (mean: 10.09, SD: 3.54) and boys (mean: 10.77, SD: 4.23) (p = 0.452). The frequency of thyroiditis by age group is shown in **Figure 2**. Among these individuals, 13 (16%) had a family history of autoimmune diseases. Age at diagnosis of AIT did not differ by family history (p = 0.214).

Thyroid functions were evaluated at the time of diagnosis of AIT; 48 ​​cases (60%) were euthyroid, 7 cases (8.8%) had subclinical hypothyroidism, and 25 cases (31.2%) had overt hypothyroidism. In the evaluation of thyroid autoantibodies, 45 cases (56.2%) had only positive anti-TPO antibodies, 5 cases (6.3%) had only positive Anti-Tg antibodies, and 30 cases (37.5%) had both thyroid autoantibodies positive. All 44 cases with available thyroid ultrasonography records showed findings consistent with AIT. Among individuals who did not initially have overt hypothyroidism, eight developed hypothyroidism 3.6 ± 2.3 years after the diagnosis of AIT during follow-up. The median age at diagnosis of hypothyroidism in individuals with AIT was 10.8 years (range, 1.25–14.75 years).

In our study, AIT was observed in 13.2% of individuals with anti-GAD antibody positivity and 11.0% of those without. The prevalence of AIT did not differ significantly according to the presence of anti-GAD, IA-2 and anti-insulin antibodies or between individuals with a single versus multiple diabetes-specific autoantibodies (p = 0.499, p = 0.375, p = 0.220 and p = 0.532, respectively).

**Figure 3:**
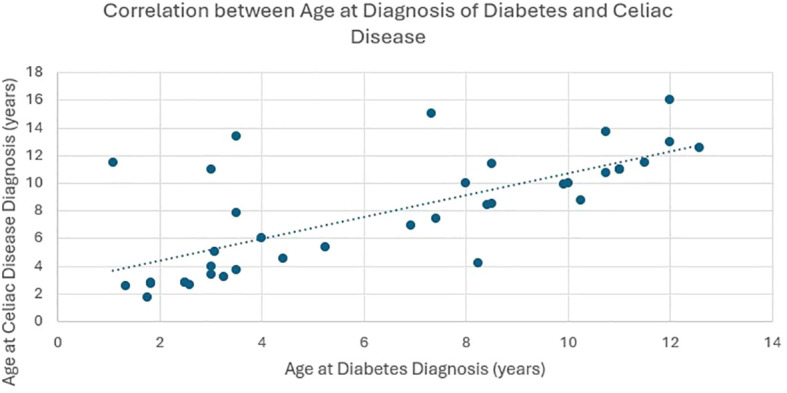
Correlation between age at diagnosis of diabetes and age at diagnosis of celiac disease.

Of the 38 cases with accompanying diagnosis of CD, 2 (5.3%) were diagnosed with celiac before diabetes, 13 (34.2) at the time of diagnosis of diabetes, and 23 (60.5%) after the diagnosis of diabetes. Of those diagnosed with CD, 20 were girls and 18 were boys. The mean age at diagnosis of CD was 7.83 ± 4.13 years. **Figure 3** illustrates the relationship between age at diagnosis of diabetes and age at diagnosis of CD. A Spearman’s rank correlation analysis revealed a strong and statistically significant positive association between age at diagnosis of diabetes and age at diagnosis of CD (ρ = 0.723, p < 0.001). This indicates that individuals diagnosed with diabetes at older ages tend to be diagnosed with CD at older ages as well.

**Figure 4:**
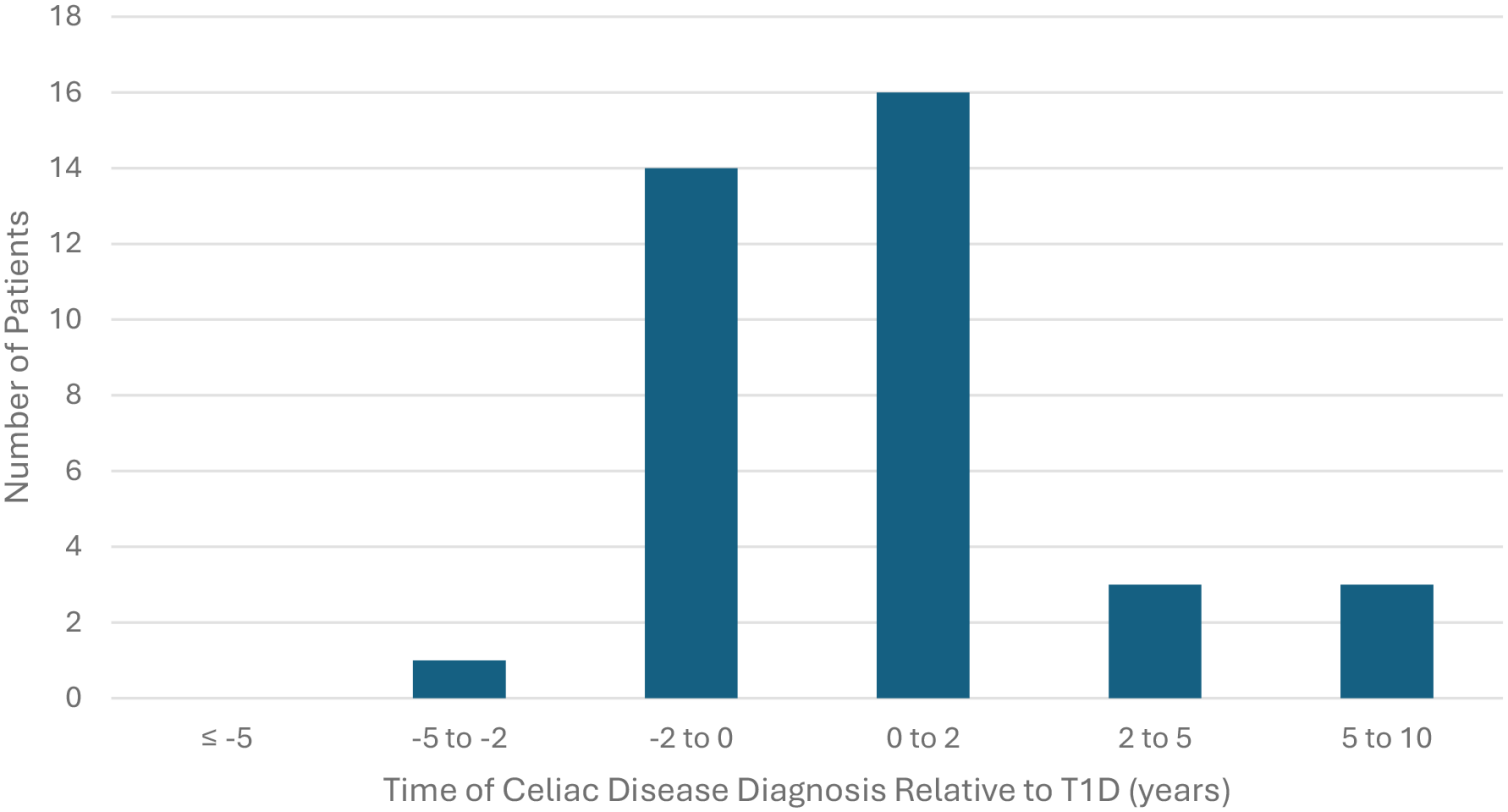
The time of diagnosis of celiac disease according to time of diagnosis of diabetes.

The mean age at diagnosis of diabetes was 6.31 ± 3.76 years in the children with CD, and 8.03 ± 3.99 years in those without. There was a statistically significant difference in the age at diagnosis of diabetes between individuals with and without CD (p = 0.012), indicating that individuals who developed T1D at a younger age had a propensity to develop CD. Among the two cases diagnosed with celiac prior to diabetes, the diagnoses were made 4.0 years and 1.5 years before diabetes. For these diagnosed with celiac after diabetes, the median time to diagnosis was 1.08 years (range, 0.16-10.4 years). **Figure 4** shows the time of diagnosis of CD according to time of diagnosis of diabetes.

In individuals diagnosed with CD, tissue transglutaminase IgA positivity was found in 26 cases (68.4%), tissue transglutaminase IgG positivity in 5 cases (13.2%), and anti-endomysium IgA positivity in 35 cases (92.1%). Anti-gliadin IgA was positive in 9 cases (23.7%), and anti-gliadin IgG in 12 cases (31.6%). Upper endoscopic biopsy results, classified according to the MARSH criteria, showed that 2 cases (5.9%) were MARSH type 1, 3 cases (8.8%) were MARSH type 2, 19 cases (55.9%) were MARSH type 3A, and 9 cases (26.5%) were MARSH type 3B. Pathology reports could not be retrieved for five individuals with biopsy-confirmed CD. All individuals with CD were following a gluten-free diet.

Among the 639 individuals with T1D, four (0.6%) had Down syndrome. Three of these individuals had at least one additional autoimmune disease, including AIT, CD, Graves’ disease, and/or vitiligo. One induvial had Turner syndrome and was diagnosed with AIT at the time of T1D diagnosis.

## Discussion

In this study, the clinical characteristics and accompanying autoimmune diseases of cases followed with a diagnosis of T1D in our center over the past 15 years were analyzed in detail. Individuals with T1D exhibit an increased prevalence of other autoimmune diseases, particularly AIT and CD. In our study, 18.8% of the cases had at least one accompanying autoimmune disease. Among the cases, AIT (12.7%), CD (5.9%), vitiligo (1.3%), autoimmune gastritis (0.15%), and AIH (0.15%) were observed. In a multicenter study conducted in Germany and Austria between 1995 and 2007, involving 31, 104 cases under the age of 18, CD-specific antibodies were positive in 11% of cases, and thyroid autoantibodies were positive in 15% (9). Taylor et al. (2011) investigated 491 children and adolescents with T1D and found AIT in 12.3%, CD in 2.8%, and Addison’s disease in one case (10). The low frequency of CD was attributed to the reluctance of many individuals with positive celiac-specific antibodies to undergo intestinal biopsy. Similarly, Doğdu et al. (2012) reported a higher prevalence of AIT and CD in children and adolescents with T1D than in healthy controls (11). In our study, consistent with the literature, the frequency of autoimmune diseases accompanying diabetes was significantly elevated.

Autoimmune diseases are known to affect females more frequently than males (12). In our study, among individuals with T1D with accompanying autoimmune diseases, 60.8% were female and 39.2% were male, with a female-to-male ratio of 1.55. Consistent with the literature (13), our multivariable logistic regression analysis confirmed that female sex was independently associated with the presence of additional autoimmune diseases, further supporting the notion that female individuals with T1D are at increased risk of developing additional autoimmune diseases.

In the general population, 1–2% of school-aged children and 6–8% of adolescents have positive antithyroid antibodies as evidence of AIT (8). In our study, thyroid-specific antibodies were positive in 12.5% of cases. The prevalence of AIT in children and adolescents with T1D has been reported to range from 11.7% to 30% in numerous studies (3, 10, 14–16). Evidence indicates that older age and longer duration of diabetes are associated with higher prevalence of AIT (17). Autoimmune thyroiditis is observed 4–6 times more frequently in females than in males, peaking in females during the pubertal age group (18). In a retrospective study conducted in our clinic, which evaluated the clinical, epidemiological, and laboratory characteristics of 162 children and adolescents diagnosed with Hashimoto’s thyroiditis, 86.4% of the cases were females and 13.6% were males, with an approximately fivefold higher prevalence in females (19). Among our individuals with T1D and coexisting AIT, 67% were females and 33% were males, indicating that the prevalence of thyroiditis was approximately twice as high in females as in males. Notably, the female predominance for AIT is less pronounced than that reported in the general population (20). This observation may reflect the underlying autoimmune environment of the T1D population, in which both sexes are at intrinsically increased risk for thyroid autoimmunity, thereby attenuating the typical gender disparity.

Of individuals with AIT, 7.5% were diagnosed a median of 1.4 years before, 42.5% at, and 50% a median of 3.4 years after their diagnosis of diabetes. Most of our individuals with T1D were found to have AIT either concurrently with or after their diagnosis of T1D. Our results indicate that, as shown in **Figure 1**, given the correlation between age at diagnosis of diabetes and age at diagnosis of AIT, thyroid screening should be performed at the time of diagnosis of T1D. This is supported by our finding that each 1-year increase in age at diagnosis of T1D was associated with a 0.594-year increase in age at diagnosis of AIT. Autoimmune thyroiditis in older children appears to occur temporally closer to the onset of diabetes. Accordingly, we propose a shorter screening interval —every 3–6 months—for individuals diagnosed with T1D at older ages.

Although our study did not reveal a significant association, several studies have demonstrated a strong link between anti-GAD antibody positivity and thyroid autoimmunity in individuals with T1D. In adult individuals with T1D, the presence of anti-GAD antibodies has been linked to a higher prevalence of thyroid-specific antibodies (21). In our study, among individuals diagnosed with AIT, 60% were euthyroid, 8.8% had subclinical hypothyroidism, 31.2% had hypothyroidism, and 1.3% had Grave’s disease. Compared to previous studies reporting higher euthyroid rates (77–81%) and lower rates of overt hypothyroidism (3–6%), our cohort showed a lower rate of euthyroidism and a higher rate of hypothyroidism (14).

Celiac disease is the second most common autoimmune disorder associated with T1D. The prevalence of CD in the general population across all age groups ranges from 0.3% to 1.0%, while among individuals with T1D it varies between 1.6% and 16.4 (4, 22). In our cohort, the prevalence of CD was 5, 9% based on antibody positivity and biopsy confirmation, consistent with previous reports, including a study from our clinic showing biopsy-confirmed CD in 5.8% of individuals with T1D (15). A study conducted by Larsson et al. in Sweden followed 300 individuals under the age of 20 with newly diagnosed T1D over a 5-year period, finding that 10% were diagnosed with CD. Among these cases, 6.8% were diagnosed prior to T1D, 34% at the time of diagnosis of T1D, and 59.2% after the diagnosis of T1D (23). Similarly, in our study, 5.3% of celiac cases were diagnosed prior to T1D, 34.2% at the time of diagnosis, and 60.5% within a median of 1.08 years afterward. In our study, most individuals (30 out of 38, 78.9%) were diagnosed with CD within a 2-year window before or after the diagnosis of T1D. In the International Society for Pediatric and Adolescent Diabetes (ISPAD) 2022 guidelines, CD screening is advised within the first year of diagnosis of T1D and every 2–5 years thereafter. However, in cases of suggestive clinical symptoms or a positive family history in a first-degree relative, more frequent evaluation is warranted due to elevated risk (24). Asymptomatic presentation is common; yet undiagnosed CD in T1D may lead to poor glycemic control, growth failure, and complications such as hypoglycemia and retinopathy (4, 25). Our findings show that despite routine annual screening, a significant proportion of individuals were diagnosed at relatively advanced histopathological stages (Marsh 3A–3B in over 80% of cases), suggesting that seroconversion and mucosal damage may have already progressed by the time of detection. Given that most diagnoses of CD in our cohort occurred within the first 2 years after T1D onset, it seems reasonable to perform screening at the time of diagnosis of diabetes and to repeat it annually during the first 2 years, when the risk of CD development is highest. Thereafter, screening at 2-year intervals appears appropriate, with earlier testing warranted if symptoms develop, growth retardation is observed, anemia appears, or thyroid or other autoimmune conditions emerge. This approach may allow for earlier detection and intervention before progression to advanced enteropathy. Overall, this approach is largely consistent with the ISPAD guidelines; however, our data underscores the importance of closer surveillance in the early years after the onset of diabetes. In addition to screening intervals, the magnitude and persistence of tissue transglutaminase -IgA positivity may also be important in deciding the timing of endoscopic biopsy in children with T1D. In a recent retrospective study including 991 children with T1D, Eviz et al. reported that tissue transglutaminase -IgA positivity was detected in 10.2% of patients, while biopsy-confirmed CD was present in 4.3%. They found that a tissue transglutaminase -IgA level ≥7 times the upper limit of normal had the best predictive value for biopsy-confirmed CD, and that fluctuating low-titer positivity may normalize spontaneously. These findings suggest that both antibody level and persistence should be considered when interpreting CD serology during follow-up (26).

Among individuals with T1D, one case of autoimmune gastritis was identified, diagnosed, and followed externally, with limited data available. Routine parietal cell antibody (PCA) screening is not performed in our clinic; thus, no data on PCA positivity is available. While studies report PCA positivity in up to 12% of individuals with T1D diagnosed before the age of 10, ISPAD 2022 guidelines recommend considering PCA testing only in cases with unexplained anemia or gastrointestinal symptoms, not for routine screening (24). The association between AIH and T1D in children is rare. A case report and literature review by Hovinga et al. (27) noted only six pediatric cases of concurrent T1D and AIH reported up to 2010. Honar et al. evaluated 202 cases of children with T1D, and among the 10 cases with positive autoantibodies, AIH was confirmed by biopsy in two cases (28). Among individuals with T1D, one case of AIH was identified. This individual, with no other autoimmune diseases, had a sibling with CD and a mother diagnosed with AIT, indicating a familial predisposition to autoimmune disorders. Although rare, AIH should be considered in the differential diagnosis of individuals with T1Dwith abnormal liver function tests due to their increased risk of autoimmunity.

In our study, 1.3% of cases were diagnosed with vitiligo. While the prevalence of vitiligo in the general population is approximately 0.5%, it is reported to be 10–20 times more common in individuals with T1D (29). The onset age for vitiligo in pediatric series is typically reported between 4 and 8 years. Since not all diabetic children and adolescents received follow-up care exclusively in our clinic, the expected higher prevalence of vitiligo compared with the general population may not have been observed. Notably, four of the eight individuals with vitiligo also had at least one additional autoimmune comorbidity, namely AIT and/or CD. Although the small number of cases precludes any firm conclusion, this observation may reflect autoimmune clustering in a subset of children with T1D and vitiligo. Therefore, the presence of vitiligo in individuals with T1D may warrant careful assessment for other autoimmune diseases during follow-up.

One of the primary strengths of this study is its large sample size, encompassing 639 children and adolescents, as well as the evaluation of the temporal relationship between the diagnosis of T1D and the occurrence of additional autoimmune diseases. The principal limitations of this study include its retrospective design and its single-center setting, which may restrict the generalizability of the findings to the broader pediatric T1D population in Türkiye or other regions. Furthermore, as not all individuals were followed exclusively at our center throughout the 15-year period, certain clinical and laboratory data may be incomplete or missing from records. Although the study spanned 15 years, some individuals had short follow-up durations, limiting our ability to assess the long-term development of autoimmune diseases. Diabetes-specific autoantibody testing was conducted both within and outside our center, which may have introduced variability due to different testing platforms. Another important limitation is the absence of longitudinal metabolic and treatment-related data, including serial HbA1c values, insulin treatment modality, insulin dose requirements, BMI trajectory, and continuous glucose monitoring metrics. Therefore, we could not evaluate the clinical impact of concomitant autoimmune diseases on diabetes management, glycemic control, treatment burden, or metabolic outcomes. Accordingly, our findings should be interpreted primarily as descriptive epidemiological data on the frequency, spectrum, and timing of autoimmune comorbidities in children and adolescents with T1D, rather than as evidence of their direct effects on diabetes-related outcomes.

This study highlights the high prevalence of autoimmune comorbidities, particularly AIT and CD, among children and adolescents with T1D. Regular screening and long-term follow-up are essential for the early detection and management of these conditions. Temporal analyses showed that most comorbidities clustered within the first two years around diabetes onset, underscoring the need for closer surveillance early in the disease course. Our findings suggest that intensified screening for AIT and CD should particularly focus on the first two years following T1D diagnosis, especially in female individuals. Our study provides valuable epidemiological data from Türkiye, supporting global comparisons and contributing to improved clinical care for pediatric individuals with T1D.

## Data Availability

The raw data supporting the conclusions of this article will be made available by the authors, without undue reservation.
